# Hemophagocytic lymphohistiocytosis associated with HBV‐HCV coinfection in adult: Case report

**DOI:** 10.1002/ccr3.4328

**Published:** 2021-08-06

**Authors:** Mounia Bendari, Hanane Delsa, Nouama Bouanani, Rajaa Jabouri, Safaa Darouich, Sara Nejjari, Fadila Guessous, Kamal Doghmi

**Affiliations:** ^1^ Hematology Department Cheikh Khalifa International University Hospital Casablanca Morocco; ^2^ Mohammed VI University of Health Sciences Casablanca Morocco; ^3^ Gastroenterology and Hepatology Department Cheikh Khalifa International University Hospital Casablanca Morocco; ^4^ Internal Medicine Department Cheikh Khalifa International University Hospital Casablanca Morocco; ^5^ Department of Biological Sciences Faculty of Medicine Casablanca Morocco

**Keywords:** HBV‐HCV co‐infection, Hemophagocytic lymphohistiocytosis

## Abstract

The case reported is the first case in Morocco to our knowledge. The reason for sharing this case is to facilitate knowledge transfer between physicians, caring for adult patients with HLH, with the aim to improve the outcome of these patients.

## Graphical abstract

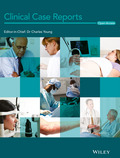

## 1 INTRODUCTION

Hemophagocytic lymphohistiocytosis (HLH) is a rare but fatal disease that is histopathologically characterized by the activation of macrophages and hemophagocytosis in bone marrow and reticuloendothelial systems. The research progress made in the HLH field raised awareness, allowing the implementation of well‐coded protocol for a better management of such condition.

Herein, and to the best of our knowledge, we report the first rare case of a woman with secondary HLH to hepatitis B virus (HBV) and hepatitis C virus (HCV) coinfection in Morocco.

Besides the treatment for hepatitis, the patient also underwent a specific therapy for HLH according to HLH‐94 protocol, which resulted in general condition improvement, transfusion independence, and splenomegaly resolution.

Our report also stresses the main concepts and characteristics of the HLH as well the current consensus and international guidelines for better clinical outcomes of these patients.

Hemophagocytic lymphohistiocytosis (HLH), a rare and fatal condition, has been subjected to a very significant revival and extensive research in recent years, proven by substantial reports and publications. Such progress raised awareness on how to establish a well‐coded protocol for a better management of such disease.

HLH is the overstimulation of the immune system leading to systemic inflammation, cytokine storm, and multi‐organ failure.^1^ It is an heterogeneous group that can be divided into two categories: primary hemophagocytic lymphohistiocytosis (HLH) and secondary hemophagocytic syndromes.^2^

Herein, and to the best of our knowledge, we report the first rare case of a woman with secondary HLH to hepatitis B virus (HBV) and hepatitis C virus (HCV) coinfection in Morocco. Upon antiviral treatment and chemotherapy, the patient achieved a complete remission.

This work prompts us to focus on more research and collaborative work among physicians and aims at sharing information about similarities and differences between cases, to improve the overall prognosis and management of such condition.

## 2 CASE REPORT

We report a rare observation about a middle‐aged woman, who is 47 years old, and she was initially referred to our hospital from Central Africa, where she was diagnosed with epigastralgia persisting for a few weeks associated with anemic syndrome and deterioration of the general state and persistent fever. The initial physical examination findings revealed fever of 40°C without signs of infection, pulse of 115/minutes, respiratory rate of 20/minutes, blood pressure of 110/60 mmHg, pallor with frank cutaneous and mucosal jaundice as well as abdominal distension with hepatosplenomegaly, and important edema of the lower limbs. Vital signs and neurological examination were normal, and no oxygen was required.

Upon recent red cell transfusion, the biological assessment with complete blood count revealed pancytopenia (hemoglobin level was at 5.8 g/dL, white blood cell counts at 2.4 × 103/L, neutrophil level at 1,3 × 103/L, lymphocyte counts at 0,9 × 10^3^/L, platelet counts at 10 × 10^3^/L), C‐reactive protein level was at 88mg/L, procalcitonin was negative, and existence of hypoalbuminemia at 24 g/L. The research of tuberculosis infections was negative. The rest of the infectious workup did not show any toxoplasmosis or leishmaniasis infection, blood cultures were negative, and the rate of LDH was 2241 UI/L.

The assessment revealed a slight cytolysis, as well as signs of portal hypertension (splenomegaly, dilated portal vein) and chronic liver disease that were shown during an abdominal ultrasound.

The upper gastroduodenoscopy displayed gastric vascular ectasia, also known as “watermelon stomach” without associated esogastric varices. To assess the cause of cirrhosis, the viral serology of hepatitis B and hepatitis C was performed and it confirmed a coinfection of viral hepatitis B and hepatitis C. The viral load results from the quantitative HCV‐RNA and HBV‐DNA in the serum were, respectively, 5,61 log UI/mL (407 380 UI/mL) and 2.02 log UI/mL (104 UI/mL).

**FIGURE 1:**
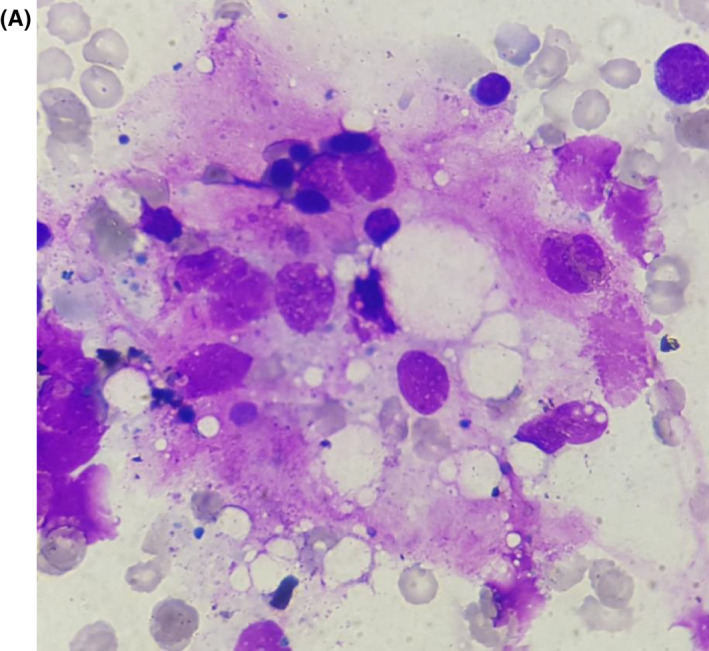
Aspiration of the bone marrow (A) reveals an increased number of activated macrophages with prominent hemophagocytosis. The hemophagocyte cell ingested red blood cells, erythroblasts, and platelets

Given the persistence of the fever and the worsening of the patient general condition in the absence of any sign of infection, a complementary assessment of febrile pancytopenia was carried out and it revealed the presence of images of hemophagocytosis at the bone marrow aspiration (Figure 1), as well as the existence of hyperferritinemia at 1535 ng/mL and hypertriglyceridemia at 3.9 g/L. On the basis of clinical and biological criteria, the diagnosis retained was the association between a macrophagic activation syndrome and an HBV‐HCV coinfection (codominant) with compensated cirrhosis.

The patient management combined the treatment for HLHs (etoposide+dexamethasone) according to the HLH‐94 protocol and antiviral therapy for HBV‐HCV coinfection with supportive care.

The HLH‐94 protocol consists of corticosteroids, typically dexamethasone daily (10 mg/m^2^ for 2 weeks, 5 mg/m^2^for 2 weeks, 2.5 mg/m^2^ for 2 weeks, then 1.25 mg/m^2^ for 1 week, and taper until it is discontinued for one week). This was followed by pulses every second week with 10 mg/m^2^ for 3 days. Associated with etoposide, the aim of the treatment is to delete activated T cells and suppress inflammatory cytokine production.

All patient characteristics are summarized in Table 1. The main complication of the treatment was corticosteroid‐induced diabetes. Treatment with etoposide was not the source of major toxicity, and side effects were mainly nausea and vomiting.

**TABLE 1:** Patient characteristics and treatment

Patient characteristic and treatment Clinical characteristics	Fever of 40°C Pulse of 115/min Blood pressure of 110/60 mmHg Cutaneous and mucosal jaundice Hepatosplenomegaly
Biological examination	Hemoglobin : 5.8 g/dL White blood cells : 2.4 × 10^3^/L Neutrophils : 1,3 × 10^3^/L Lymphocytes : 0,9 × 10^3^/L Platelets : 10 × 10^3^/L Hypertriglyceridemia : 3.9 g/L Hyperferritinemia : 1535 ng/mL LDH : 2241 UI/L. Hemophagocytosis at the bone marrow aspiration

The antiviral therapy was based on the European Association for the Study of the Liver (EASL) Guidelines and was mainly adapted to the available drugs in Morocco: sofosbuvir +daclatasvir for 6 months for hepatitis C and tenofovir for hepatitis B.

After 3 months of treatment, the evolution was favorable with apyrexia, resolved cytopenia, and total disappearance of jaundice. The patient general condition was clearly improved with regression of transfusion needs until transfusion independence, normalization of the biological balance, and undetectable viral load for both viruses.

## 3 DISCUSSION

In 1979, Risdall et al were the first to describe a new clinical entity characterized by fever, hepatosplenomegaly, and pancytopenia. Lymphadenopathy, rash, and lung damage were often present. In this first description, it was also observed that the bone marrow and the lymph nodes were infiltrated by macrophages of normal morphology, which phagocytized erythrocytes, platelets, and leukocytes.^3^ The autopsy of six fatal cases showed subarachnoid and hepatic infiltration with hepatocyte necrosis. It was the first precise description of hemophagocytosis syndrome.

In recent years, the pathophysiology of HLH has become well understood thanks to the study of primary forms. It was acknowledged that HLH is due to a activation and/or cytotoxicity defect of CD8 T cells and NK cells, which produce large amounts of interferon γ that lead to an activation of macrophages in the bone marrow and reticuloendothelial cells, which in turn produce pro‐inflammatory cytokine.^4^

As a result, plasma levels of pro‐inflammatory cytokines IL‐1, IL‐6, TNF‐α, IFN‐γ, M‐CSF, and IL‐18 were elevated.

They are roughly divided into primary hemophagocytic lymphohistiocytosis (HLH) and secondary hemophagocytic syndromes. Primary HLH is the consequence of genetic mutations altering the cytotoxic function of the natural killer (NK) and cytotoxic T cells, which are generally found in infancy and childhood. In addition, familial HLH (F HLH) affects patients who have autosomal recessive mutations affecting perforin (FHLH2), MUNC 13‐4 (UNC13D), STX11 (coding for syntaxin 11), and STXBP2 (coding for syntaxin‐binding protein 2).^5^, ^6^ It also includes other inherited immunodeficiency syndromes with hypopigmentation such as Chédiak‐Higashi syndrome, Griscelli syndrome, and type II Hermansky‐Pudlak syndrome.^7^ On the other hand, secondary HLH usually affects adolescents and adults, but it is considered as a reactive syndrome. Most cases of secondary HLH are associated with “predisposing condition” causing immune dysregulation, such as malignancy (particularly lymphoma), immunodeficiency, or autoimmune disease, and/or a “trigger,” most commonly infection such as Epstein‐Barr virus (EBV).^8^ Among adults, infections represent the most prevalent triggers of HLH, with increasing age, and other causes can be found such as malignancies, primarily lymphomas.^9^, ^10^ As for our patient, the clinical presentation was concordant with infectious disease, and the assessment of fever and jaundice. The etiological assessment showed a viral hepatitis B and hepatitis C infection; however, despite the antiviral treatment, the persistence of the fever made us carry out more assessment at the research of HLH, which turned out to be positive.

In fact, the diagnosis of HLH among adults must be done according to the HLH‐2004 diagnostic criteria within clinical judgment and the patient's history.

In 1991, the Histiocyte Society suggests a standardized set of five diagnostic criteria for HLH used for the prospective HLH‐94 clinical trial. Such criteria were revised and validated in 2004, and the new standardized version included more diagnosis criteria and stated that, for a positive diagnosis of HLH, individuals need to meet five out of eight diagnostic criteria.^11^, ^12^

The diagnosis of HLH can be confirmed if a molecular diagnosis consistent with HLH, or the presence of the five following criteria: fever, splenomegaly, cytopenias (affecting 2 of 3 lineages in the peripheral blood), hemoglobin less than 90 g/L (hemoglobin less than 100 g/L in infants, 4 weeks), platelets less than 100 Giga/L, neutrophils less than 1 Giga/L, hypertriglyceridemia and/or hypofibrinogenemia, fasting triglycerides more than 3.0 mmol/L (ie, more than 265 mg/dL), fibrinogen more than 1.5 g/L, hemophagocytosis in bone marrow or spleen or lymph nodes, no evidence of malignancy, low or no NK cell activity (according to local laboratory reference), ferritin more than 500 mg/L, and sCD25 (ie, soluble IL‐2 receptor) more than 2400 U/mL.^13^

Other diagnosis tools that are not part of the HLH‐2004 criteria consist of hyperbilirubinemia, hepatomegaly, transaminitis (usually found among patients with HLH), and elevated lactate dehydrogenase and D‐dimer levels, with the latter elevated on the majority of patients even when international normalized ratio, partial thromboplastin time, and fibrinogen are normal. These tools listed above proved useful to distinguish between HLH, septic shock, and conditions such as autoimmune hemolytic anemia. They can also help as a response for a therapy evaluation.

For our patient, all criteria for HLH were present; she was febrile and had a splenomegaly, cytopenia, hyperferritinemia, hypertriglyceridemia, and specific images of hemophagocytosis. Additionally, she was admitted to our unit with sepsis and pancytopenia, and therefore, exploring the presence of peripheral hemophagocytosis has been proposed quickly for identifying adult secondary HLH.

Viral hepatitis is considered as the leading cause of chronic liver disease worldwide and the first cause of cirrhosis in Africa. However, the hepatitis B virus (HBV) and hepatitis C virus (HCV) coinfection is a complex clinical entity that has an estimated worldwide prevalence of 1‐15%.^14^ Most clinical studies have shown that the progression of the disease is faster in HBV‐HCV–coinfected patients compared to those with monoinfection, with high rates of decompensated cirrhosis^15^ and increased incidence of hepatocellular carcinoma.^16^ For our patient, the hepatitis B and hepatitis C coinfection was considered as the cause of HLH, as this viral infection could be the origin of T‐cell activation and inflammatory cytokine production. Treatment in coinfected patients is complex, especially due to the interaction of the two viruses.

Until now, there are no clear treatment guidelines for HBV‐HCV coinfection. But many studies showed good results with the new drugs such as direct‐acting antiviral agents.^17^ This treatment should be discussed by all candidates for chemotherapy and immunosuppressive therapy with active viral hepatitis, especially hepatitis B.

The first‐line therapy in adults with infection‐triggered HLH consists of antimicrobials. In these cases, the role of chemotherapy and immunosuppression is not clear. Sometimes, systemic steroids can be added to antimicrobials, but its benefits are unknown due to lack of data.^18^ In our case, besides the treatment for hepatitis, the patient also underwent specific therapy for HLH according to HLH‐94 protocol,^19^ which resulted in general condition improvement, transfusion independence, and splenomegaly disappearance.

## 4 CONCLUSION

Despite its low incidence, HLH is considered as a fatal disease and should be brought up quickly in the presence of any unexplained prolonged fever.

In our case, HLH was associated with an HCV‐HBV coinfection, which is very rare. The early concomitant treatment of viral hepatitis infection and HLH proved efficient for our patient.

All physicians should be aware of HLH because its early recognition may prevent irreversible organ damage and subsequent death. They should not hesitate to realize the biological assessment and to repeat it if necessary to confirm the presence of HLH in order to be able to start the specific treatment in time, and thus improve the prognosis, which remains bleak for this pathology.

Overall, the aim of this case report is to share our experience with other fellow physicians caring for adult patients with HLH combined with viral infection and to improve their clinical outcome.

## CONFLICT OF INTEREST

All authors declare no competing interests.

## AUTHOR CONTRIBUTIONS

Mounia Bendari: acts as a principal and correspondent author, and has played an important role in redaction and literature searching. *Hanane Delsa: participated equally with a principal author in writing. Nouama Bouanani: acts as co‐author and has carried out a revision of the text. Rajaa Jabbouri: participated in literature searching. Safaa Darouich and Sara Nejjari: have equally taken part in drafting of the article. Fadila Guessous: participated in correcting and revising of the article. Kamal Doghmi: has played a substantial role in designing the article.

**DATA AVAILABILITY STATEMENT**

The data that support the findings of this study are available on request from the corresponding author. The data are not publicly available due to privacy or ethical restrictions.
